# Unilateral Cochlear Implantation Reduces Tinnitus Loudness in Bimodal Hearing: A Prospective Study

**DOI:** 10.3389/fneur.2017.00060

**Published:** 2017-03-07

**Authors:** Jérôme J. Servais, Karl Hörmann, Elisabeth Wallhäusser-Franke

**Affiliations:** ^1^Department of Otorhinolaryngology, Cochlear Implant Centre, University Medicine Mannheim, Mannheim, Germany; ^2^Audiology, Medical Faculty Mannheim, Department of Otorhinolaryngology, Heidelberg University, Mannheim, Germany

**Keywords:** tinnitus, loudness reduction, cochlear implant, bimodal hearing, anxiety, depression, prospective study

## Abstract

Perceptive and receptive aspects of subjective tinnitus like loudness and tinnitus-related distress are partly independent. The high percentage of hearing loss in individuals with tinnitus suggests causality of hearing impairment particularly for the tinnitus percept, leading to the hypothesis that restoration of auditory input has a larger effect on tinnitus loudness than on tinnitus-related distress. Furthermore, it is assumed that high levels of depression or anxiety prevent reductions of tinnitus loudness and distress following restoration of activity in the cochlea. This prospective study investigated the influence of unilateral cochlear implant (CI) on tinnitus in 19 postlingually deafened adults during 6 months following implantation. All had bimodal provision with the other ear being continuously supported by a hearing aid. On the day before CI implantation (T1, T2), and at about 3 and 6 months postsurgery (T3, T4), participants were questioned about their current tinnitus. Loudness was rated on a Numeric Rating Scale, distress was assessed by the TQ12 Tinnitus Questionnaire, and depression and anxiety were recorded with the Hospital Anxiety and Depression Scale. At T2, 79% experienced tinnitus, one participant developed tinnitus after implantation. Following implantation, tinnitus loudness was reduced significantly by 42%, while reductions in tinnitus-related distress (−24%), depression (−20%), and anxiety (−20%) did not attain statistical significance. Significant correlations existed between tinnitus measures, and between postimplantation tinnitus-related distress and anxiety and depression scores. Moreover, improvement of hearing in the CI ear was significantly correlated with reduction in tinnitus loudness. A new aspect of this study is the particular influence of CI provision on perceptive aspects of preexisting tinnitus (hypothesis 1), with the effect size regarding postimplant reduction of perceived tinnitus loudness (1.40) being much larger than effect sizes on the reduction of tinnitus-related distress (0.38), depression (0.53), and anxiety (0.53). Contrary to expectation both tinnitus measures reduce even in the majority of CI recipients with increased levels of anxiety or depression. This suggests that reduction of the tinnitus signal by restoring activity in the cochlea cannot be entirely compensated for by central tinnitus mechanisms and results in a reduction of perceptive and less so of reactive aspects of subjective tinnitus.

## Introduction

This prospective study addresses changes in subjective tinnitus following cochlear implantation. A new aspect is the investigation of bimodal implantees, who hear with the help of a cochlear implant (CI) on one ear and an acoustic hearing aid (HA) on the contralateral ear. This combination of hearing substitution is rather common and provides significant real-world benefit as compared to unilateral CI (1). Moreover, influence on perceived loudness of the tinnitus and tinnitus-related distress are assessed separately and effect sizes are calculated.

Hearing loss is a major risk factor for tinnitus (2), and therefore, it is not surprising that tinnitus is common among CI candidates. On average, as many as 80% of CI candidates experience tinnitus (3, 4). It was realized early that CIs may reduce tinnitus (5) with several authors reporting on tinnitus reduction (6–11). However, the opposite, namely, exacerbation of a preexisting tinnitus, or development of tinnitus with CI use has also been observed (7, 12). The risk of developing tinnitus following CI ranges from 0 to 4%, while worsening of a preexisting tinnitus has been reported in 1–9% of cases (7). As tinnitus may lead to considerable suffering (13–15), the circumstances influencing its suppression, versus its worsening, or even the emergence of new tinnitus with CI use need to be explored.

The tinnitus signal or percept is thought to arise in the central auditory system in response to a hearing deficit, which in most cases can be attributed to impairments in the cochlea (16). The burden experienced by tinnitus extends beyond this percept and was shown to correlate with anxiety and depression (14, 17). Heterogeneity and severity in symptoms associated with tinnitus is reflected in a wide variety of proposed treatments (13) and may be the reason why overall effectiveness of currently available treatments is suboptimal.

Tinnitus is an auditory percept that varies in persistence, can be localized to one or both ears or is heard within the head, and that is perceived with variable loudness. Beyond this, people with tinnitus may suffer from their tinnitus and the amount of suffering cannot solely be determined by the perceptive qualities of the tinnitus, rather appearing to be associated more closely with mental health (14, 17, 18). In agreement, the reactive component, or distress related to the tinnitus percept, was found to be related to alterations in the emotional, attentional, and memory systems of the brain and to altered interactions between these systems and the auditory system: reportably leading to undue salience of the tinnitus signal (19).

We want to address the following hypotheses: first, we propose that because CIs restore input into the central auditory system, CI provision has a stronger effect on perceptive aspects of tinnitus than on reactive aspects. Thus, we expect CIs to primarily reduce tinnitus permanence and perceived loudness, and possibly change its localization if the hearing balance is altered between ears. At the same time, we expect a weaker effect of CI use on tinnitus-related distress, because this aspect of tinnitus is thought to depend mainly on interactions with the non-auditory brain [e.g., Ref. (15, 19)]. Second, we propose that high levels of depression and anxiety counteract CI-induced tinnitus attenuation, resulting in a lesser reduction of tinnitus-related distress and loudness in individuals with high depression and/or anxiety scores.

Moreover, as CIs are constantly improved in terms of type of implant, electrode insertion and positioning, and speech processing strategy, greater tinnitus suppression is expected by newer types of implants (6, 7). Therefore, it is important to explore the tinnitus reducing capacities of currently available CI technology.

The aims of this prospective study are therefore to (i) estimate the change of perceptive and (ii) reactive tinnitus measures separately, and (iii) examine the influence of mental health on the reduction of tinnitus symptoms following unilateral CI implantation in bimodal users.

## Materials and Methods

### Procedure and Inclusion

Between 2014 and 2016, study participants were recruited from the patients at the CI Centre of the University Medical Centre Mannheim. Prospective participants had postlingual onset of profound hearing impairment. Recruitment was independent of reported tinnitus. Inclusion criteria comprised: first-time unilateral CI provision, an Advanced Bionics (HiRes 90K) implant as chosen by the patient, HA use at the other ear, and aged between 18 and 90 years. All patients who fulfilled these criteria were approached for inclusion. Exclusion criteria were assessed during an initial interview (T1) and included: insufficient knowledge of the German language, more than mild cognitive deficit, as assessed by the DemTect Test (20), and use of other implanted devices. Twenty-five patients were included in the study. Two participants discontinued the study following sequential bilateral implantation, one discontinued because of health conditions unrelated to the study or their tinnitus, one decided that study participation after T2 was too much effort, and two discontinued for reasons they did not disclose.

The initial interview, study inclusion (T1), and presurgery examination (T2) took place on the same day, usually the day before surgery (mean [SD]: 3 [7] days). Patients received a CI on their weaker ear while HA use was continued on the other ear. They left hospital on average 3 days postsurgery. Two to three weeks later, they participated in a week-long in-patient program with first fitting of the speech processor, several fitting sessions, and technical instruction on CI use. Until the first formal appointment at the CI Centre of the University Medical Centre Mannheim, 4 weeks following surgery, participants’ mean daily processor use was 11 h. Postimplantation assessments T3 and T4 were scheduled for 3 and 6 months postimplantation (T3: 100 [18] days; T4: 221 [70] days).

Before T1, all subjects gave written informed consent in accordance with the Declaration of Helsinki. The protocol was approved by the Medizinische Ethikkommission II of the Medical Faculty Mannheim (approval no. 2014-527N-MA). Study participants were compensated for their participation.

### Participant’s Characteristics

Etiology of hearing loss varied greatly and was unknown in many cases. The decision which ear was to be implanted was based on various audiological and anatomical criteria and was generally independent of reported tinnitus. Nineteen subjects completed the study. Before implantation, four did not experience tinnitus and three of them did not report tinnitus at any assessment, while one developed tinnitus between T2 and T3.

Study participants focused much more on their hearing problems than their tinnitus. When asked at pre-assessment (T1) what they expected of their CI, none mentioned tinnitus. No expectations were expressed by one participant, whereas the remaining 18 expected their hearing to improve in order to understand spoken language more easily and to participate in social situations again. Further characteristics of study participants are given in Table 1.

**Table 1:** **Participant’s characteristics**.

	Tinnitus at T2	No tinnitus at T2
Number	15	4
Gender: male (N)	2	2
Age mean (SD)	55.3 (14.0)	64.3 (19.3)
Cochlear implant (CI) left	9	2
CI ear: years with hearing impairment	25.5 (18.5)	24.0 (13.0)
Hearing aid (HA) ear: years with hearing impairment	21.3 (19.6)	17.0 (11.8)
Pre-OP HA use CI ear (excluding cross)	10	3
Pre-OP HA use other ear	15	4
PTA-4 (dB HL) of CI ear		
Preimplantation	95.9 (17.3)	91.3 (15.0)
Postimplantation	46.6 (12.8)	43.8 (9.7)
PTA-4 (dB HL) of HA ear		
Preimplantation	65.5 (18.6)	70.1 (15.1)
Postimplantation	61.1 (24.0)	72.3 (4.5)
Hospital Anxiety and Depression Scale (HADS) with at least 1 scale ≥8 at T2	6	0
Participants with relevant other health conditions	9	3

*Shown are numbers per condition and means with respective SDs. PTA-4 thresholds of the CI ear decrease significantly with implantation indicating much better hearing postimplantation. Although the group without tinnitus is older, duration of hearing impairment at the HA ear is shorter, and hearing on the HA ear is worse, differences between tinnitus and non-tinnitus group are not statistically significant. Another potential difference between those affected by tinnitus and those without may be differences in mental wellbeing. Scores of 8 points or above in the HADS are seen as indicator of potential problems regarding depressiveness and anxiety, respectively, and are present only in the tinnitus group. Other relevant health conditions were, for instance, hypertension, thyroid, cardiologic, depression, and condition following a malignant tumor*.

### Measures

Pure tone audiometry (PTA) was conducted in sound field for both ears at 0.5, 1, 2, and 4 kHz both prior to and postsurgery with HA and if applicable CI. Measures across these frequencies were averaged separately for CI and HA ears (PTA-4). If a response could not be obtained because a frequency was not heard by the participant, values were set to 120 dB HL, i.e., 10 dB above the highest sound presentation level used during audiometry. The same questionnaires and audiological tests were used at the assessments preceding implantation (T2), as well as approximately 3 (T3) and 6 (T4) months postsurgery.

In addition to general background and expectations, questions about the presence, persistence, and location of tinnitus, a Numeric Rating Scale (NRS) (21) assessing current subjectively perceived tinnitus loudness (NRS 0–10: tinnitus audible only in silence—tinnitus louder than all other sounds) were used. Additionally, tinnitus-related distress was assessed with the 12-item version of the Tinnitus Questionnaire [TQ12, named MTQ in Ref. (14, 15)]. The TQ12 was developed by Hiller and Goebel (22) according to an optimal combination of high item-total correlations, reliability, and sensitivity for the assessment of changes in tinnitus-related distress. According to Zeman et al. (23), the TQ showed satisfying psychometric results, which were equally good for the long form and for the short TQ12 form. Internal consistency of TQ12 was α = 0.87 (23). For a classification of tinnitus-related distress, Hiller and Goebel (22) proposed four grades with: scores 1–7 (grade 1) signifying no clinically relevant distress, a score of 8–12 (grade 2) representing moderate distress, a score of 13–18 (grade 3) representing severe distress, and a score of 19–24 representing the most severe distress due to tinnitus. Grades 3 and 4 are considered to require therapeutic intervention. Depression and anxiety were assessed with the Hospital Anxiety and Depression Scale [HADS (24)] at T2 and T4. For both subscales of the HADS, scores of 8 or above are considered to be indicative of potential problems in these areas (24). For tinnitus patients, internal consistencies of α = 0.83 and α = 0.88 were determined for the anxiety and depression subscales (25). One question with five options between “very good” (4) and “poor” (0) assessed the subjective impression of a participant regarding his/her general health condition at T2 and T4.

### Statistical Analysis

Statistical analysis was performed with IBM SPSS Statistics, version 22 (SPSS/IBM, Chicago, IL, USA). Descriptive statistics included mean and SD. The effect of intracochlear electrical stimulation on tinnitus was assessed by comparing baseline values to the outcomes obtained at the end of the follow-up. Pearson correlation coefficients were calculated, with coefficients <0.5 being considered as weak, coefficients between 0.5 and 0.8 being considered as moderate, and >0.8 being considered as strong. In addition, a general linear model for repeated measurements with Bonferroni correction and in case of non-normality Friedman tests were performed prior to *post hoc* testing with two-sided *t*-tests or Wilcoxon signed-rank tests, respectively. Statistical significance was defined for *p* values smaller than 0.05, and *p*-values smaller than 0.01 were considered to be highly significant.

Effect sizes for repeated measures in dependent samples were calculated according to Bortz (26) [see also Ref. (27)] with the following equation:

$$\varepsilon =\frac{\mu_{1}-\mu_{2}}{\hat{\sigma }\sqrt{1-r}}$$

The difference between means (μ_1_, μ_2_) is divided by the pooled variance $\left(\hat{\sigma }\right)$. Introduction of the Pearson correlation coefficient (*r*) serves as a correction for the dependence among means. Effect sizes of >0.2 correspond to weak effects, of >0.5 to moderate, and of >0.8 to strong effects.

## Results

### Pure Tone Audiometry

Preoperative aided thresholds at 0.5, 1, 2, and 4 kHz (PTA-4) in free sound field could not be determined for 10 of the 24 CI ears and for 1 HA ear due to no response in some or all of these frequencies. For calculation of overall improvement of hearing, values for measurements that did not yield a result were set to 120 dB HL. Pre- and postimplantation PTA-4 averages are shown in Table 1. Improvement of hearing thresholds of CI ears with CI use was highly significant (*t* = 10.593; *p* < 0.001), while average thresholds in the HA ear were unchanged (*t* = 1.078; *p* = 0.301). For participants with preexisting tinnitus, the CI ear remained the worse ear for three, became the better hearing ear for seven, and aided hearing levels were similar for both ears in five individuals. For all individuals without tinnitus, the CI ear became the better ear postsurgery.

### Tinnitus before CI

Prior to implantation, tinnitus was reported by 79% (15/19) of the study participants. The four participants without tinnitus were older, duration of hearing impairment on the other ear was shorter, preimplantation hearing thresholds were more similar for the two ears, and they were less likely to report high scores in the anxiety and depression scales. Differences to the tinnitus group were not statistically significant, however. In contrast, reported duration of hearing impairment on the ear to be implanted was similar to the average in the tinnitus group (Table 1).

**Figure 1:**
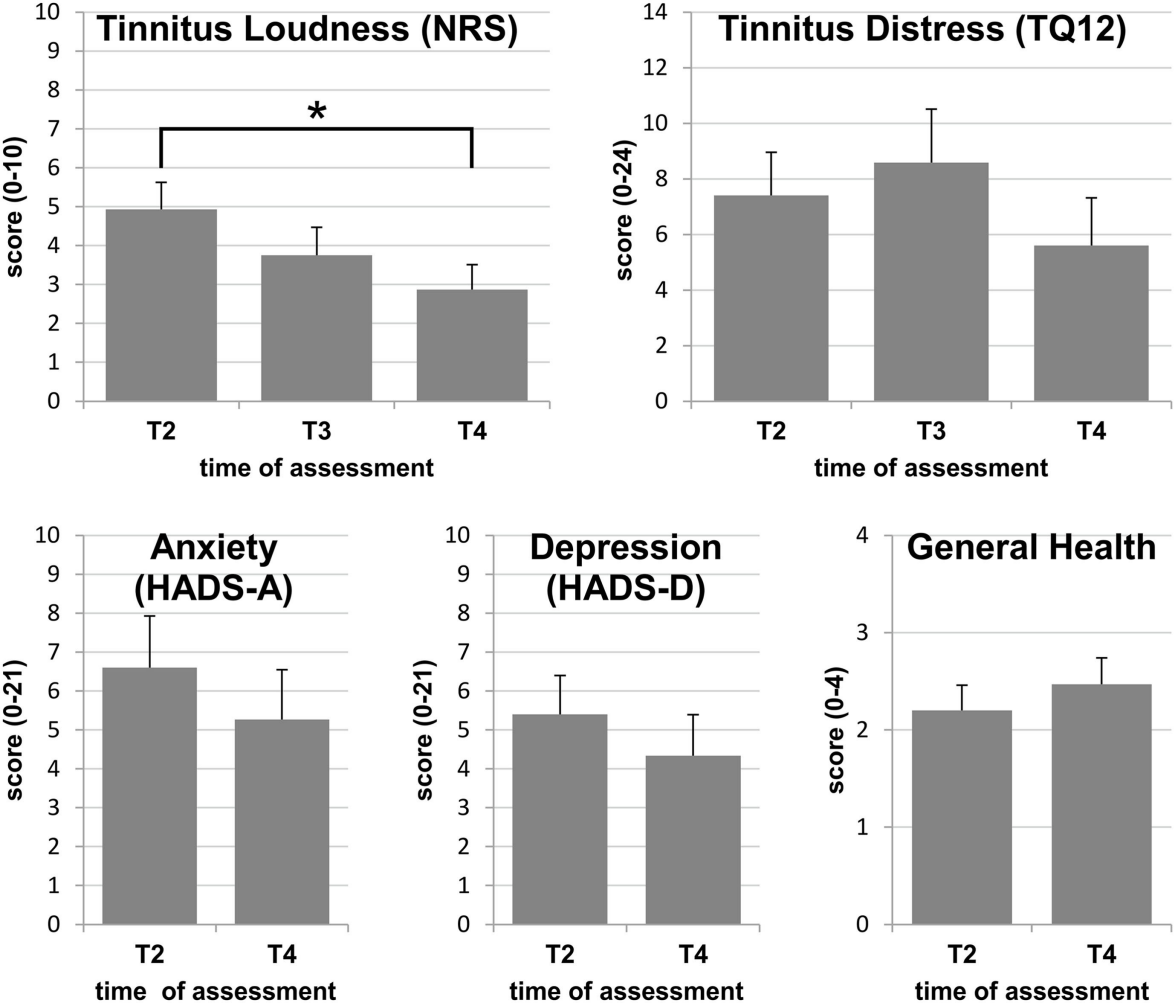
**Group means with SEs at assessments T2, T3, and T4: only the reduction of perceived tinnitus loudness reaches statistical significance, while differences between assessments did not attain statistical significance for tinnitus-related distress, anxiety, depression, or general health**. Statistically significant differences are indicated by *p < 0.05.

Preexisting tinnitus was permanent in 7 cases, and was localized across both ears, or was heard within the head in 10 individuals, while it localized to the future CI ear in 4 and to the HA ear in 1. Perceived loudness was very loud (≥8) for four participants and very low (≤2) for 2, with an average of 4.9 [(2.7), range 1–10] on the 0–10 NRS scale. According to the grading of Hiller and Goebel (22) with TQ12 scores above 12 indicating clinically significant tinnitus-related distress, two participants (13%) expressed clinically significant tinnitus-related distress, while the average score was 7.4 [(6.0), range 1–24], corresponding to low tinnitus-related distress (see Figure 1).

### Changes in Tinnitus with CI Use

Of the 15 study participants with tinnitus at T2, all but 2 reported a subjective benefit following CI use. In two subjects with tinnitus prior to surgery, an additional tone arose for the CI ear or the HA ear, respectively. One subject developed new tinnitus at the CI ear, which was neither loud or distressing and not permanent by T4. At T2, seven participants reported permanent tinnitus, while this was true for six at T4.

Changes in tinnitus localization after cochlear implantation coincided with improvement of PTA-4 thresholds in the CI ear relative to the HA ear. Whereas tinnitus localization did not change if the CI ear remained the worse ear (*n* = 3), it changed in 50% of the other 12 in whom hearing balance between CI and HA ear was changed by CI use.

For calculations of average scores for the tinnitus and health variables, values obtained from the individual with tinnitus onset after surgery were omitted. On average, tinnitus loudness decreased by 42% between T2 and T4 resulting in an effect size of 1.40, which indicates a strong effect (Figure 1). Overall, this reduction was highly significant (*F* = 9.161; *p* = 0.012), and the reduction between T2 and T4 reached significance with *post hoc* testing (*p* = 0.035). Tinnitus loudness was at least halved in 47%. At the end of the study, mean perceived loudness was 2.9 (2.5) and was rated as 5 or below for all except one subject (Figure 1). This subject experienced a tinnitus of maximal loudness (10/10) at onset and at the end of the study.

On average, TQ12 scores decreased by 24% between T2 and T4 resulting in an effect size of 0.38 which indicates a weak effect of bimodal provision on tinnitus-related distress (Figure 1). The main effect just missed statistical significance (chi^2^ = 5.911; *p* = 0.052), while *post hoc* tests clearly missed a significance level (T2–T3: *Z* = −0.996; *p* = 0.319; T2–T4: *Z* = −1.646; *p* = 0.100). Between T2 and T4, the TQ12 score decreased by at least 3 points (12.5% on the 0–24 TQ12 scale) for eight participants (53%), was unchanged for 5 (33%), and increased by at least 3 points for two participants. The latter both indicated severe stress independent of their hearing at the end of the study. At T4, all but three participants had tinnitus of the lowest category, grade 1, and one fell into grade 2, indicating mild to moderate tinnitus (Figure 1). The remaining two participants did not benefit from CI use in terms of tinnitus-related distress reduction. One of them reported maximal tinnitus-related distress (24/24) both at the beginning and at the end of the study, coinciding with maximal tinnitus loudness (10/10) at both assessments. This participant was diagnosed with an additional attack of sudden sensorineural hearing loss on the non-implanted ear during the study and expressed a high level of anxiety and depression symptoms at all times. The other participant had very low tinnitus loudness and distress prior to CI surgery. With CI use, tinnitus loudness increased from 2 to 3 points on the NRS, whereas the TQ12 score was increased by 17 points at T3 and by 16 points at T4, as compared to pre-CI, reaching a level of severe tinnitus distress (grade 3) at the post-CI assessments. Noteworthy in this participant were the continuously high levels of depression and anxiety. This was despite taking antidepressive medication since T3, prescribed independently of the study.

### Anxiety, Depression, and General Health

On average, the levels of anxiety and depression were both reduced by 20% with an effect size of 0.53 indicating moderate effects on both factors (Figure 1). Reductions did not reach significance for either factor (anxiety: *t* = 1.451; *p* = 0.169; depression: *Z* = −1.307; *p* = 0.191). At the first assessment prior to implantation, six of the participants with preexisting tinnitus (40%) reported a score of 8 or above, either in one or in both HADS subscales, i.e., indicating potential problems in these areas. At T4, three participants reported a score of ≥8 in one of the HADS scales. During the study, scores dropped below 8 in 4 participants and increased in one, whose initial scores had been inconspicuous. The latter case could be related to events independent of the CI. Despite indications of mental health problems, tinnitus loudness and distress were reduced by more than 50% in four of the six participants who reported increased HADS scores at T2, and also in the individual whose HADS scores increased above 8 during the study.

When asked for a judgment of their general health situation, only one individual reported a substantial improvement of 3 points on the 0–4 scale between T2 and T4. On average, general health was considered to be satisfactory at T2, and the majority did not report any improvement, often despite a statement that their hearing ability had improved considerably (Figure 1).

### Correlation between Measures

In the correlation matrices presented in Tables 2 and 3, correlations between subjective tinnitus loudness and tinnitus-related distress peaked between significant to highly significant, but moderate correlations were found at all assessments. In addition, correlations between postimplantation tinnitus-related distress and the level of anxiety at T2 and T4 and also with the level of depression at T2 exhibited significant to highly significant correlations of moderate strength. When correlating changes during the study period, a significant correlation existed between improvement of hearing at the CI ear and the reduction of tinnitus loudness between T2 and T4 (*r* = 0.584; *p* = 0.022*). In addition, time of hearing impairment showed a significant correlation with the improvement in anxiety scores between T2 and T4 (Table 3). Age at implantation did not show a significant correlation with any of the above variables, but a significant inverse correlation with general health indicating that older participants had more health problems. According to the reporting of chronic health conditions by the participants, these were mostly unrelated to their hearing (Table 1).

**Table 2:** **Correlations between measures**.

	T2-TNRS	T3-TNRS	T4-TNRS	T2-TQ12	T3-TQ12	T4-TQ12	T2-HADS-A	T4-HADS-A	T2-HADS-D	T4-HADS-D	T2-Health	T4-Health
Age at implantation	0.044	0.206	0.055	0.253	0.195	0.027	0.068	0.239	0.295	0.083	−0.246	−0.647
	0.875	0.521	0.846	0.363	0.544	0.925	0.810	0.392	0.285	0.768	0.376	0.009**
T2-TNRS	1	0.589	0.697	0.576	0.528	0.274	−0.038	0.146	0.040	−0.121	−0.546	−0.139
		0.044*	0.004**	0.025*	0.078	0.322	0.893	0.603	0.889	0.667	0.035*	0.622
T3-TNRS		1	0.785	0.826	0.767	0.624	0.139	0.383	−0.009	−0.077	−0.043	−0.147
			0.002**	0.001**	0.004**	0.030*	0.666	0.247	0.978	0.813	0.894	0.649
T4-TNRS			1	0.780	0.760	0.756	0.375	0.482	0.237	0.089	−0.472	−0.220
				0.001**	0.004**	0.001**	0.168	0.069	0.395	0.752	0.075	0.432
T2-TQ12				1	0.651	0.495	0.256	0.477	0.059	−0.176	−0.178	−0.244
					0.022*	0.061	0.357	0.072	0.835	0.529	0.526	0.381
T3-TQ12					1	0.851	0.587	0.672	0.510	0.498	−0.421	−0.537
						<0.001**	0.045*	0.017*	0.090	0.100	0.172	0.072
T4-TQ12						1	0.681	0.710	0.558	0.508	−0.411	−0.458
							0.005**	0.003**	0.031*	0.053	0.128	0.086
T2-HADS-A							1	0.725	0.772	0.654	−0.365	−0.524
								0.002**	0.001**	0.008**	0.180	0.045*
T4-HADS-A								1	0.582	0.616	−0.197	−0.643
									0.023*	0.014*	0.482	0.010**
T2-HADS-D									1	0.814	−0.541	−0.648
										<0.001**	0.037*	0.009**
T4-HADS-D										1	−0.258	−0.482
											0.354	0.069
T2-Health											1	0.505
												0.055

*Significant correlations exist between subjective tinnitus loudness and tinnitus-related distress. Further significant correlations are observed for postimplantation tinnitus-related distress with anxiety at T2 and T4 and with depression scores at T2. Furthermore, tinnitus loudness and depression at T2 and anxiety scores at T2 and T4 as well as age at implantation show significant inverse correlations with general health*.

Pearson correlation coefficients: **p < 0.01 (2-tailed); *p < 0.05 (2-tailed).

*HADS-A, Hospital Anxiety and Depression Scale for anxiety; HADS-D, Hospital Anxiety and Depression Scale for depression*.

**Table 3:** **Correlations between changes in measures with cochlear implant (CI) use**.

	Pre- to postimplantation improvement					
	CI ear: PTA-4	TNRS	TQ12	Hospital Anxiety and Depression Scale for anxiety (HADS-A)	Hospital Anxiety and Depression Scale for depression (HADS-D)	Health
Age at implantation	−0.239	−0.008	0.211	−0.160	0.389	−0.422
	0.391	0.977	0.451	0.568	0.151	0.117
CI ear: years of hearing impairment	−0.338	−0.315	−0.250	0.580	−0.073	0.220
	0.218	0.253	0.368	0.023*	0.796	0.431
Hearing aid (HA) ear: years of hearing impairment	−0.466	−0.380	−0.319	0.613	−0.129	−0.058
	0.080	0.163	0.246	0.015*	0.647	0.839
CI ear: PTA-4 improvement	1	0.584	0.354	−0.273	−0.191	0.424
		0.022*	0.195	0.325	0.496	0.115
TNRS improvement		1	0.405	−0.312	−0.025	0.231
			0.134	0.258	0.929	0.408
TQ12 improvement			1	−0.364	0.082	−0.002
				0.182	0.772	0.994
HADS-A improvement				1	0.442	0.246
					0.099	0.377
HADS-D improvement					1	0.113
						0.688

*Significant correlations are present between improvement of PTA-4 thresholds pre- to postimplantation of the CI ear and improvement, i.e., reduction of subjective tinnitus loudness between T2 and T4, and between years of hearing impairment of the CI and the HA ear and the reduction in anxiety scores between T2 and T4*.

Pearson correlation coefficients: *p < 0.05 (2-tailed).

## Discussion

Tinnitus was common for the hearing-impaired participants of this study. Main findings are a significant reduction in subjective tinnitus loudness between preimplantation and 6 months postimplantation (T2 versus T4), and a significant correlation between improvement of hearing with CI use and the reduction in tinnitus loudness. As predicted by hypothesis 1, restoration of activity in the cochlea had a stronger effect on subjective tinnitus loudness, and less influence on tinnitus-related distress. Moreover, magnitude of influence on tinnitus-related distress was lower than effects on anxiety and depression. Contrary to hypothesis 2 that high levels of anxiety or depression prevent reduction of tinnitus symptoms, these reduced in the majority of participants with high depression and anxiety scores, while failure to reduce or increases could be related to other current sources of distress.

To the best of our knowledge, this is the first longitudinal study on the influence of bimodal provision on tinnitus symptoms. Present findings are in general agreement with findings on unilateral electric amplification of sound by CI as reported in retrospective studies (11, 28–30), reviews (3, 4, 31–33), and a growing body of prospective studies [e.g., Ref. (7, 12, 34–39)]. Although total remission from tinnitus was not observed in the present study, most subjects noticed substantial reduction of their tinnitus, while worsening of a preexisting tinnitus was rare, predominantly pertained tinnitus-related distress, and appeared to be associated with increased levels of anxiety and depression. Emergence of tinnitus only after cochlear implantation was an exception, and as reported before (7, 12), resolved within a few weeks and was experienced as mild at the end of follow-up. In the sample by Pan et al. (12), those who acquired tinnitus had the shortest duration hearing loss and were the oldest implant recipients. This cannot be corroborated by the present results, however.

The effect size for loudness reduction indicates a strong effect comparable to or higher than effect sizes that were reported for generally accepted tinnitus therapies that, however, serve to reduce tinnitus-related distress as opposed to tinnitus loudness (40, 41). Two prospective studies on tinnitus following cochlear implantation addressed tinnitus loudness in a similar way, namely, by a visual analog scale (35, 38). The participants of these studies had severe to profound unilateral or bilateral hearing impairment and were provided unilaterally with a CI while hearing was not amplified at the other ear. Tinnitus loudness was reduced significantly and by a similar amount as in the present study, but effect sizes for the reduction of tinnitus loudness were not reported in these publications.

Bimodal provision was far less effective in reducing tinnitus-related distress. This finding was expected since tinnitus-related distress depends on further influences that cannot directly be influenced by restoration of cochlear activity (19). Furthermore, presurgery tinnitus-related distress was reported as mild to moderate by the majority of the study participants, even if the tinnitus was rather loud. This is in line with earlier studies on CI implantees (36, 39) and may have prevented findings of significant reductions. Taken together, present findings support the assumption that restoration of auditory input primarily reduces the tinnitus signal, whereas it has a weaker influence on tinnitus-related distress, and they support the distinction between perceived tinnitus loudness and distress (14, 15, 42). Furthermore, these findings corroborate the assumption that tinnitus-related distress is influenced by non-auditory factors as suggested previously (15, 17–19, 43).

Acquired hearing impairment represents a risk factor for increased levels of anxiety and depression (44, 45), especially in combination with distressing tinnitus (14, 18). A total of 40% of those with preexisting tinnitus indicated conspicuous levels of anxiety and/or depression before implantation, whereas average levels were low which is in accordance with earlier results from CI recipients [e.g., Ref. (28, 29, 35, 36)]. Depression and anxiety scores were reduced by 20% between baseline and the end of follow-up, but these differences did not attain statistical significance. Former reports differ in this aspect with some observing significant reductions whereas others do not, with discrepancies likely being dependent on sample characteristics (28–30, 36). Estimated magnitude of effects for the reduction of anxiety and depression were higher than for the reduction in tinnitus-related distress. This suggests that other aspects of life quality related to anxiety and depression may have been improved by bimodal provision (44, 45). Correlations of postimplant levels of tinnitus-related distress with anxiety and for fewer comparisons also with depression attained significance. An association between these measures has been reported for tinnitus populations with and without cochlear implants (8, 14, 28), and catastrophic interpretations of tinnitus have been associated with fear (46). But increased levels of anxiety or depression did not prevent a reduction in tinnitus loudness and tinnitus-related distress, except in two participants, indicating that a reduction in tinnitus is highly reliant on afferent auditory input. Despite a reduction in tinnitus-related complaints and better hearing in general, individuals with higher scores in the HADS scales were not satisfied with their quality of life. Although low in terms of percentage, CI recipients who do not experience tinnitus relief, or express compromised well-being, need to be taken care of as they might benefit from other types of therapies (13), and this in turn might improve performance with their CI.

The exact mechanism through which CI use suppresses tinnitus symptoms is unknown. Several mechanisms, such as masking, direct electrical nerve stimulation, habituation, and plastic reorganization in the brain have to be considered. Another aspect why CIs appear to be effective in reducing the tinnitus may be the intense auditory training required during rehabilitation. According to current knowledge, tinnitus is the result of maladaptive plasticity in the central auditory pathway in response to auditory deprivation. In the majority of cases, the trigger for tinnitus-related changes in the brain is impairment of the cochlea (16). Experimentally inducing auditory deprivation by exposing healthy subjects to complete silence triggers phantom sounds that are reversible upon restoration of the auditory input (47). In addition, continuous use of earplugs can lead to a reversible perception of tinnitus (48). This suggests that tinnitus can be induced by auditory deprivation and that it can be reversed by restoring input into the central auditory system. Restoration of peripheral input especially at the base of the cochlea which is important for activity in the tinnitus-relevant high-frequency areas of the auditory system can be achieved by CI use or by other types of electrical stimulation (8). Extracochlear (49–51) and intracochlear electrical stimulation (52–54) reduce tinnitus, even when the stimuli are “not audible.” A study by Punte et al. (55) suggested that tinnitus suppression does only occur if the full length of the cochlea is electrically stimulated by a CI. On the other hand, continued presence of tinnitus during CI use may be due to the fact that hearing is not completely restored by the implant, or because a memory of the tinnitus has been established in the brain which is partly independent of external input.

Although electrical stimulation of the inner ear effectively suppresses tinnitus for many CI recipients, the reported percentages vary between studies and may depend on characteristics of the samples under study, particularly regarding non-auditory aspects (29). For conscious perception of the tinnitus signal and for the suffering arising from it, involvement of brain areas beyond the auditory system appears to be mandatory (19). Thus, tinnitus is not simply the result of defective auditory input but obviously requires further mechanisms. This may be the reason why some that are hard of hearing, or deaf, do not experience tinnitus: for instance, 21% of the participants of the present study. Brain regions with alterations related to tinnitus have included the emotional, attentional, and memory systems. These systems are thought to influence processing in the auditory cortex and thus in the auditory system in a top-down manner (19, 56), particularly in those who suffer from their tinnitus (19, 57, 58). Behaviorally, this is evidenced for instance by enhanced levels of depression and anxiety in tinnitus populations especially, among those with distressing tinnitus (14, 15). Remarkably and similar to other studies (28, 35), average depression and anxiety scores are low in the present CI sample. This may be a favorable condition for allowing a reduction of the tinnitus signal in a bottom-up manner and may part explain the extent of tinnitus reduction by CIs. Although tinnitus with a major emphasis on non-auditory mechanisms (19) was thought to respond less to recovery of the auditory input, even individuals with enhanced levels of anxiety and depression responded to bimodal provision with a substantial reduction of tinnitus symptoms, given that there existed no other current sources of severe distress. In the latter, the tinnitus might even worsen despite attenuation of its trace within the auditory system. Noteworthy in this respect are the two individuals whose tinnitus was not positively influenced by bimodal hearing. Whereas in one, the tinnitus retained maximal loudness and distress throughout the study period, in the other negligent tinnitus-related distress before CI provision increased tremendously with CI use. Concomitantly high levels of anxiety and depression at pre- and postsurgical assessment in both individuals and the reporting of severe distress postimplantation are in agreement with the assumption that highly distressing tinnitus may predominantly be a consequence of central top-down processing and that it is therefore, less influenced by attenuation of the tinnitus signal in the ascending auditory pathway.

Taken together, CIs alone as well as in combination with a contralateral HA appear to be effective in reducing the tinnitus signal through influencing neurophysiological processes involved in the generation and maintenance of tinnitus, via compensation of peripheral deafferentation. In addition, enhanced attentiveness to environmental sounds following implantation may lower awareness of the tinnitus. Although psychological factors certainly contribute to the tinnitus relief obtained through implant use, restoring auditory input by electrical stimulation appears to be primary effect. In accordance, loudness is reduced to a larger extent than the tinnitus-related distress. Whether long-term CI use reverses reorganization in the central auditory system associated with peripheral deafferentation and with tinnitus (16) remains to be shown. Tinnitus reduction does not always result in increased well-being, however. In our study sample, individuals with increased levels of anxiety or depression still felt anxious or depressed, despite improvements in their tinnitus and their hearing in general. Such individuals might additionally require psychological help.

### Limitations

As in all prospective studies with CI recipients, the sample under investigation is small and therefore, might not be representative. However, our results are in general agreement with the published literature. Study participants were observed in their first 6 months after cochlear implantation. This time may be too short as an endpoint since improvements in auditory comprehension tend to continue thereafter. However, others (35, 38) have shown that reductions of tinnitus loudness occur early after CI provision. Furthermore, as double-blind studies are not feasible in this patient group, this was an open study that is not completely free from bias. It is conceivable, however, that placebo-controlled studies are and will be exceptional in intracochlear electrical stimulation for tinnitus suppression. For our sample, it can be stated that, while amelioration of tinnitus was not the main focus of the participants’ concerns or expectations, most experienced relieving tinnitus reduction.

## Conclusion

Restoration of auditory input by bimodal provision (CI and contralateral HA) appears to be an efficient method of reducing tinnitus: primarily the perceived loudness. At the same time, the risk of worsening or developing tinnitus as a result of implant surgery is low. Therefore, restoration of auditory input in the high-frequency part of the cochlea, as achieved by a CI, can be regarded as an effective means for the reduction of tinnitus, but is only justified in patients with deeply compromised hearing ability. Electrical stimulation of the cochlea independent of CI use might represent a feasible alternative for those with better hearing yet experiencing loud tinnitus.

In addition to an effective stimulation of the tinnitus-relevant high-frequency range of the cochlea, and consequently of central auditory structures, effectiveness of CI use regarding tinnitus reduction might also be related to the low levels of tinnitus-related distress, depression, and anxiety commonly found prior to implantation. Based on our findings, we propose that central mechanisms exacerbating tinnitus have to be expected in individuals who exhibit increased levels of anxiety and depression in particular if they experience severe distress postimplantation. Although those who suffer from their tinnitus and show compromised mental health represent a small percentage of CI recipients with tinnitus, this aspect has to be taken care of. The impact on quality of life, possibly on acceptance of the CI, means that these individuals may require other types of specialized therapeutic interventions, interventions which however, are available (13).

## Author Contributions

JS: data acquisition, interpretation of data, and drafting of the manuscript. KH: critical revision. EW-F: study conception and design, data acquisition, analysis and interpretation of data, and drafting of the manuscript.

## Conflict of Interest Statement

The authors have the following interests. This study was partly funded by Advanced Bionics AG, Staefa. Switzerland. Advanced Bionics AG manufactures the device under investigation in this study. This does not alter the authors’ adherence to all the Frontier policies as detailed online in the guide for authors.
